# Benchmarking Quantum Solvers in Noisy Digital Simulations for Financial Portfolio Optimization

**DOI:** 10.3390/e28080916

**Published:** 2026-08-14

**Authors:** Ruizhe Shen, Zichang Hao, Ching Hua Lee

**Affiliations:** Department of Physics, National University of Singapore, Singapore 117542, Singapore; zichang.hao@u.nus.edu (Z.H.); phylch@nus.edu.sg (C.H.L.)

**Keywords:** quantum simulation, variational quantum algorithms, quantum portfolio optimization, quantum approximate optimization algorithm

## Abstract

In this work, we benchmark two prominent quantum algorithms: Quantum Imaginary-Time Evolution (QITE) and the Quantum Approximate Optimization Algorithm (QAOA) for obtaining the ground state of Ising-type Hamiltonians. Specifically, we apply them to the Markowitz portfolio optimization problem in quantitative finance, on both digital quantum computers and local quantum simulators with controllable two-qubit errors (noise). In noiseless settings, we find that QAOA achieves excellent convergence to the optimal results. Under noisy conditions, the QITE method exhibits greater robustness and stability, though it incurs substantially more classical numerical cost. In contrast, we demonstrate that QAOA offers better scalability and can still yield robust results if the noise can be effectively mitigated. Our findings provide valuable insights into the trade-offs between scalability and noise tolerance and demonstrate the practical potential of quantum algorithms for solving real-world optimization problems on near-term quantum devices.

## 1. Introduction

Leveraging quantum computing for real-world applications represents a promising yet challenging frontier [1,2,3,4,5,6,7,8]. With its ability to process and manipulate information with qualitatively heightened efficiency, quantum processors have significant potential to outperform classical resources in solving complex problems across domains from physics [5,9,10,11,12,13,14,15,16,17,18,19,20,21,22,23,24,25,26,27,28] to practical applications such as cryptography and drug discovery [29,30,31,32,33,34]. More recently, quantum computing has demonstrated substantial promise in tackling computationally demanding optimization problems [35,36,37,38,39,40]. A particularly relevant example with practical importance is portfolio optimization in quantitative finance [41,42,43,44]. This task involves constructing a portfolio that maximizes expected returns, a problem traditionally addressed using classical optimization algorithms and machine learning techniques [43,44,45,46]. As the complexity and dimensionality of modern financial portfolios increase, quantum hardware is emerging as a compelling tool for managing sophisticated optimization models.

To solve such optimization problems on digital quantum computers, one first needs to formulate them within a quantum Hamiltonian framework, such that the optimal solution corresponds to the ground state of a spin-1/2 system. A widely used method is Quantum Approximate Optimization Algorithm (QAOA) [35,36,47,48,49,50,51,52]. This approach is implemented through optimizing a parameterized circuit and minimizing the expectation value of a cost function. Beyond these methods, Quantum Imaginary-Time Evolution (QITE) provides a deterministic route to ground-state preparation by evolving a quantum state along imaginary time [53,54,55].

Previous studies have applied QAOA and related quantum optimization methods to portfolio selection using mean–variance, QUBO, and knapsack-based formulations [41,42,56,57]. In particular, Huot et al. formulated portfolio selection as a knapsack problem and developed a QAOA-based approach aimed at improving the quality of feasible portfolio solutions [57]. These studies establish QAOA as a viable framework for financial portfolio optimization, and the present work does not claim its first application in this context. The contribution of our work is instead a noise-aware benchmark of QAOA and QITE applied to the same discretized Markowitz portfolio Hamiltonian. We compare the two methods in terms of energy convergence, portfolio-return error, optimal-bitstring identification, output-state distributions, noise sensitivity, and circuit-level resource requirements. QAOA is examined in both noiseless and controlled-noise simulations, while the compiled QITE circuits are additionally implemented on IBM superconducting quantum hardware. This common framework allows us to identify their distinct practical limitations: QAOA achieves accurate convergence under ideal conditions but becomes unstable when noisy cost-function estimates enter its iterative optimization loop, whereas QITE retains identifiable optimal solutions under the finite-size hardware conditions studied here, at the cost of deeper circuits and substantial offline variational compilation. Our study, therefore, complements previous QAOA-based portfolio-optimization works by focusing specifically on the noise robustness and computational trade-offs between two different quantum ground-state preparation strategies.

This work is organized as follows: In Section 2, we introduce the problem of financial portfolio optimization and the potential advantages of tackling it through a quantum algorithm. Following this, we introduce the Markowitz portfolio optimization problem and detail its mapping to a quantum Hamiltonian. In Section 3, we describe the quantum optimization algorithms employed in this study, including QAOA and QITE. Then, in Section 4, we present simulation results and compare the performance of QAOA and QITE under both noiseless and noisy conditions. Finally, we summarize our benchmarking results of the two quantum algorithms and discuss applications for future research in Section 5.

## 2. Portfolio Optimization Problem

In the financial sector, there exist a variety of optimization problems [58,59,60,61]. These include forecasting tasks such as pricing, risk assessment, anomaly detection in transactions, and identifying customer preferences, as well as optimization tasks like selecting optimal portfolios, developing effective trading strategies, and designing robust hedging techniques. Portfolio optimization specifically refers to the problem of identifying the optimal allocation of a given set of assets [43,44,62]. In this section, we provide an introduction to the problem of Markowitz portfolio selection.

### 2.1. Markowitz Portfolio Selection

Here, we first introduce the mathematical model for Markowitz portfolio selection [63,64,65,66]. In this framework, a fixed budget *b* can be allocated among *m* assets. Each asset *u* is characterized by its expected return $r_{u}$. The model also incorporates a covariance matrix $c_{u,v}$, which quantifies the historical price correlation between assets *u* and *v*. Here, the task of Markowitz portfolio selection is to construct an optimal portfolio that maximizes expected return while minimizing overall portfolio variance. This formulation is based on Markowitz portfolio theory, which aims to achieve an efficient trade-off between risk and return [43].

Next, we introduce the quantized minimum investment fraction $p_{w}=1/2^{w-1}$, where w∈N parametrizes the number of budget slices $2^{w-1}$. The investment fraction for the *u*-th asset can be described as an integer multiple $z_{u}p_{w}$, where the integer $z_{u}$ indicates how many quantized units are allocated to asset *u*. We then impose the constraint that the total investment must match the full budget: $\sum_{u}z_{u}p_{w}=1$. Within this framework, the Markowitz portfolio selection problem is reformulated as an optimization task: identifying the optimal set of $z_{u}$ values that maximizes the following objective function [43]

(1) $$F\left(z\right)=\theta_{1}\sum_{u=1}^{m}r_{u}z_{u}-\theta_{2}\sum_{u,v=1}^{m}c_{u,v}z_{u}z_{v}-\theta_{3}\sum_{u=1}^{m}{\left(z_{u}p_{w}b-b\right)}^{2}.$$

Here, *b* represents the real amount of budget, while the product $z_{u}p_{w}$ ($0\leq z_{u}p_{w}\leq 1$) denotes the fraction of the budget allocated to asset *u*. The parameters $\theta_{1,2,3}$ represent the investor’s preferences with respect to expected return, portfolio risk, and tolerance for budget deviation, respectively. Specifically, the $\theta_{1}$ term rewards high expected return, the $\theta_{2}$ term penalizes risk arising from asset correlations, and the $\theta_{3}$ term imposes a penalty for deviation from the total budget [43]. The inclusion of the factor *b* in the $\theta_{3}$ term aligns it with the budget dependence of $r_{u}$ (Equation (2)) and $c_{u,v}$ (Equation (3)) in the first two terms. This ensures that the budget deviation penalty remains comparable in magnitude to the return and risk terms, even when *b* is large. In our implementations, we set $\theta_{1}=0.8$, $\theta_{2}=0.1$, $\theta_{3}=0.1$, and b=10. More details are presented in Appendix B.

### 2.2. Correspondence Between Financial and Quantum Models

To solve this optimization problem described by Equation (1) on a quantum platform, we first map it onto the ground-state problem of a corresponding quantum Hamiltonian. To achieve this, we rescale the expected return based on historical price data and define

(2) $$r_{u}=\frac{bp_{w}}{a_{u,N_{f}}}\cdot \sum_{l=1}^{N_{f}}\frac{a_{u,l+1}-a_{u,l}}{N_{f}-1},$$

where $a_{u,l}$ is the *l*-th history of price data for the *u*-th asset and $N_{f}$ denotes the number of historical assets price points [67,68]. In this work, these historical prices are randomly generated, and details are provided in Appendix B. The corresponding covariance matrix $c_{u,v}$ is computed as

(3) $$c_{u,v}=\frac{b^{2}p_{w}^{2}}{a_{u,N_{f}}a_{v,N_{f}}}\cdot \frac{\sum_{l=1}^{N_{f}}\left(a_{u,l}-{\bar{a}}_{u}\right)\left(a_{v,l}-{\bar{a}}_{v}\right)}{N_{f}-1}.$$

where $\bar{a}$ denotes the mean price of assets. To enable encoding these data on a qubit system, we represent each integer $z_{u}$ in binary form:

(4) $$z_{u}=\sum_{k=1}^{w}2^{k-1}x_{i(u,k)},$$

where i(u,k)=(u−1)w+k and $x_{i}\in \left\{0,1\right\}$ is a binary variable indicating the presence or absence of investment in a specific quantized unit.

For instance, the optimal bitstring shown in Figure 6 is 010100100 (101011011 under little-endian qubit ordering). This solution encodes an investment configuration for a portfolio of three assets with three binary slices per asset. The actual investments are $z_{0}/p_{w}=1/2$, $z_{1}/p_{w}=1/4$, and $z_{2}/p_{w}=1/4$.

Under this representation, the original optimization problem is reduced to finding the ground state of an Ising Hamiltonian:

(5) $$H=\sum_{i}h_{i}\sigma_{i}^{z}+\sum_{i,j}J_{i,j}\sigma_{i}^{z}\sigma_{j}^{z}+\delta$$

where classical binary quantities are mapped to quantum operators via the transformation $x_{i}\to \frac{1}{2}\left(1+\sigma_{i}^{z}\right)$, with the Pauli operator $\sigma_{i}^{z}$. The coefficients $h_{i}$, $J_{i,j}$ and δ can be derived from the parameters appearing in Equation (1), which are computed from the asset prices, as detailed in Appendix A. The quantity δ is independent of the quantum state and shifts all energy levels uniformly. It, therefore, does not affect the optimal bitstring or the optimization results and is omitted in our numerical calculations. The coupling matrix $J_{ij}$ is formally dense because it contains contributions from both the asset covariance matrix and the global budget constraint. However, analysis of the released coupling matrices shows that the data-dependent inter-asset long-range contribution is small, accounting for less than approximately 0.4% of the total coupling norm in the examined instances. The dominant long-range interaction originates from the budget constraint and has a highly structured, low-rank form. Consequently, the Hamiltonians considered here are structured dense portfolio QUBOs rather than nearest-neighbor models or generic fully connected spin-glass models with strong random frustration.

There exists previous works that demonstrated how this portfolio optimization problem can be addressed using quantum methods [40,42,43,44,52,69,70,71,72]. However, most focus on noiseless settings, which do not accurately reflect the limitations of noise in current platforms, including gate errors, decoherence, and imperfect measurements. Given these, we implement and benchmark different quantum algorithms on noisy simulators and demonstrate whether these methods can identify optimal results under certain controlled noise levels. In the following section, we will provide a comprehensive discussion of these quantum algorithms and their effectiveness in optimizing financial portfolios.

## 3. Methods

In this section, we outline the methods employed to solve the optimization problem described above using digital quantum processors and their simulators.

### 3.1. Quantum Optimization Algorithms

The Quantum Approximate Optimization Algorithm (QAOA) is a variational algorithm that can be employed to approximate the ground state of a given Hamiltonian [35,69]. This method operates by minimizing the expectation of the target Hamiltonian $H_{p}$ through a parameterized quantum circuit. Generally, this is achieved by minimizing the cost function

(6) $$C^{0}\left(s\right)=\langle \Psi \left(s\right)|H_{p}|\Psi \left(s\right)\rangle ,$$

but we later introduce a modification to this form in order to accelerate convergence. Here, s is a generalized vector containing the entire set of variational parameters $\left(s_{0},s_{1},\ldots \right)$, where each $s_{i}$ denotes the angles in each rotational gate, as defined in Equation (9). These variational angles are optimized by minimizing the difference between the exact ground-state energy $E_{g}$ and the estimated energy E(s), i.e., by minimizing the cost function

(7) $$C\left(s\right)=|E_{g}-E\left(s\right)|.$$

**Figure 1:**
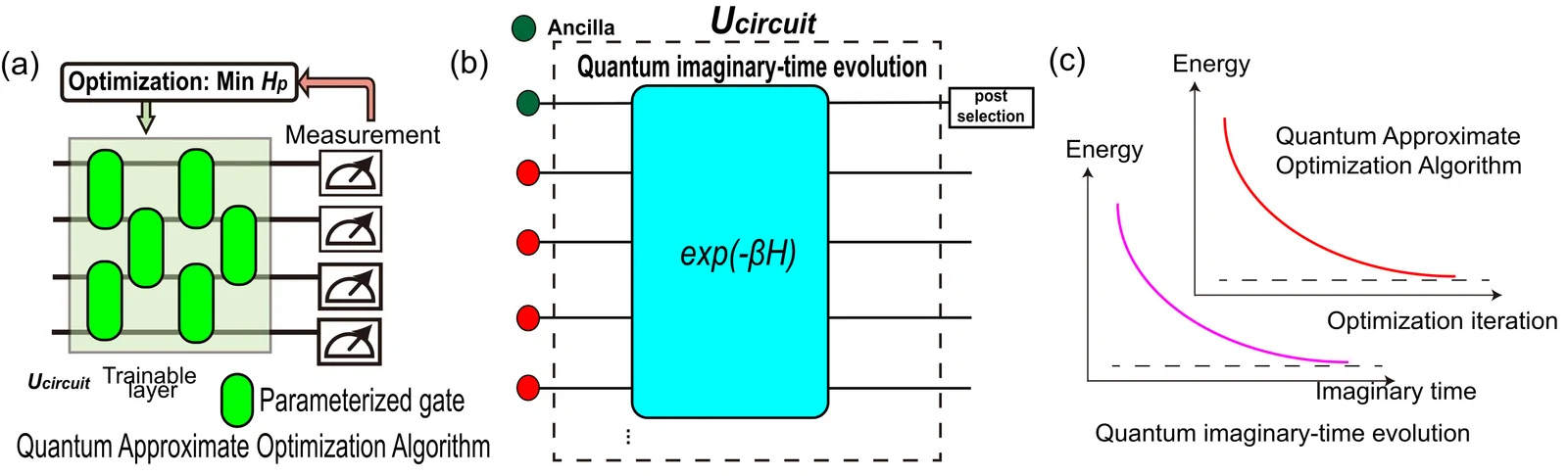
Schematics of the Quantum Approximate Optimization Algorithm (QAOA) and Quantum Imaginary-Time Evolution (QITE) approaches. (**a**) The QAOA framework, where a parameterized quantum circuit is iteratively optimized to minimize the expectation value of the target Hamiltonian $H_{p}$. (**b**) Quantum circuit structure for implementing QITE. The green qubit represents the ancilla qubit, and the red qubits correspond to the system qubits used for encoding the problem instance. The system qubits are coupled to the ancilla qubit to implement the QITE dynamics, $e^{-\beta H}$. Here, *H* represents the Hamiltonian defined in Equation (5), whose ground state is to be found. The final result is obtained by post-selecting the ancilla qubit in the |0〉 state. Additional implementation details are provided in Appendix D. (**c**) Evolution of QITE and QAOA. The energy of both systems decreases under imaginary time (violet curve) or optimization iterations (red curve) to approximate ground states (dashed line).

This setup is schematically shown in Figure 1a. Note that $E_{g}$ is the exact ground level, which is a relative energy shift. In general, this term is not strictly necessary, since QAOA can be implemented by directly minimizing the cost function defined in Equation (6). However, incorporating the relative shift often accelerates convergence, thereby reducing the number of iterations and lowering the overall simulation cost.

To simulate this optimization process on quantum circuits, we first evaluate the expectation value E(s), obtained by measuring the expectation of each local term in the Hamiltonian. We then iteratively adjust the parameters in s to minimize the deviation $|E_{g}-E\left(s\right)|$. After each update, the new parameters are re-uploaded to the quantum circuit, and the cycle of measurement and optimization is repeated until the cost function reaches convergence within a reasonable tolerance. For the classical optimization step, we primarily employ the COBYLA (Constrained Optimization BY Linear Approximations) optimizer to achieve optimization [73]. As an alternative, we also test the L-BFGS-B (Limited-memory Broyden–Fletcher–Goldfarb–Shanno with Box constraints) algorithm [73]. While both methods are viable, we consistently observe that COBYLA achieves faster and more reliable convergence in practice.

Through an alternating evolution process, QAOA efficiently explores the Hilbert space to approximate the ground state. The specific structure of the variational circuit used in this work is detailed in Section 3.3. While QAOA has demonstrated strong effectiveness and versatility across a variety of optimization tasks, its performance is not robust in the presence of noise in tasks that require high-fidelity identification of optimal quantum states, as we will demonstrate below. In the following section, we conduct a detailed evaluation of QAOA performance in solving this problem, with a particular focus on its robustness in noisy environments and its effectiveness in approximating optimal quantum states.

### 3.2. Quantum Imaginary-Time Evolution

We next discuss the Quantum Imaginary Time Evolution (QITE) method. This approach obtains the ground state of a quantum system via the following evolution:

(8) $${|\psi \rangle }_{\mathrm{ground}}\approx e^{-\beta H}|\psi (0)\rangle /\sqrt{\left|\right|e^{-\beta H}|\psi (0)\rangle \left|\right|},$$

where *H* is the system Hamiltonian, and β is the imaginary time parameter [53,54]. As β increases, excited states are suppressed, and the evolution converges towards the ground state of the system. Since this process is intrinsically non-unitary, its implementation on quantum hardware requires an effective embedding strategy: the non-unitary operator $e^{-\beta H}$ is realized through an extended unitary operation which we denote as $U_{\mathrm{circuit}}$, as schematically illustrated in Figure 1b. In this setup, an ancilla qubit (shown in green) is coupled to the physical qubits to implement the QITE process. Then, post-selection on an ancilla qubit enables the extraction of the desired state $e^{-\beta H}|\psi (0)\rangle /\sqrt{\left|\right|e^{-\beta H}|\psi (0)\rangle \left|\right|}$. Further technical details are provided in Appendix D.

### 3.3. Variational Circuits for Implementing QAOA and QITE

**Figure 2:**
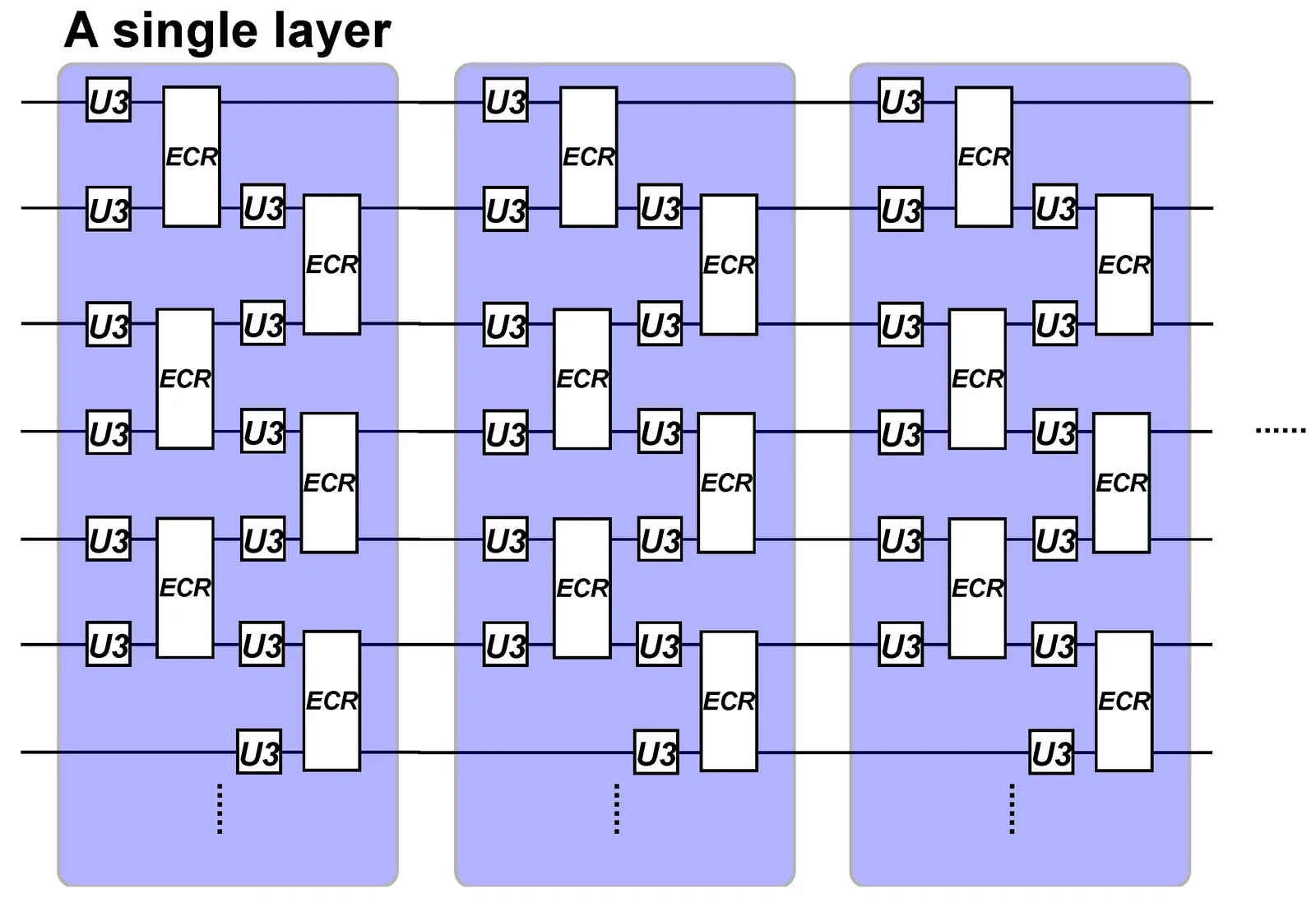
Structure of our trainable variational quantum circuits. Each layer (purple block) consists of parameterized single-qubit $U_{3}$ gates followed by ECR (Echoed Cross Resonance) gates that entangle two adjacent qubits. The optimization process is achieved through optimizing the angular parameters in each $U_{3}\left(\theta ,\phi ,\lambda \right)$ gate. For our local noisy simulation, each ECR gate is implemented through a CX gate. The entire circuit in this work consists of 2 to 8 of such variational layers, depending on the degree of optimization desired. This structure is used for the implementation of both QITE and QAOA methods.

To implement QAOA and QITE on an actual quantum circuit, we employ variational circuits, i.e., parametrized quantum circuits to be optimized. The structure of a variational circuit *V* is shown in Figure 2, where the variational parameters enter each of the $U_{3}$ gates

(9) $$U_{3}\left(\theta ,\phi ,\lambda \right)=\left[\begin{array}{cc} \cos\left(\frac{\theta }{2}\right) & -e^{i\lambda }\sin\left(\frac{\theta }{2}\right)\\ e^{i\phi }\sin\left(\frac{\theta }{2}\right) & e^{i(\phi +\lambda )}\cos\left(\frac{\theta }{2}\right) \end{array}\right],$$

which is parameterized by three tunable angles θ,ϕ,λ. The entire variational circuit *V* is thus parameterized through the collection of all the vectors $s_{i}=\left(\theta_{i},\phi_{i},\lambda_{i}\right)$.

Variational circuits are used differently in QITE and QAOA. Exactly representing the target unitary $U_{\mathrm{circuit}}$ used in the QITE process (corresponding to the circuit shown in Figure 1b) as a quantum circuit is typically infeasible, as it may involve circuit depths that are orders of magnitude greater than what is allowed by current NISQ noise thresholds. However, a properly optimized variational circuit *V* can approximate $U_{\mathrm{circuit}}$ rather accurately through the minimization of the following cost function

(10) $$\begin{array}{c} C^{\mathrm{QITE}}\left(s\right)=1-\mathrm{Tr}\left[V^{†}\left(s\right)U_{\mathrm{circuit}}\right]/2^{n}\\ =1-\langle \psi_{h}|V^{†}\left(s\right)U_{\mathrm{circuit}}|\psi_{h}\rangle \end{array}$$

where *n* is the number of qubits in the entire circuit, and $|\psi_{h}\rangle ={\mathrm{Hadamard}}^{⊗n}|000\ldots \rangle$. In the well-optimized limit where $C^{\mathrm{QITE}}\left(s\right)\approx 0$, we have $V\approx U_{\mathrm{circuit}}$.

For the QAOA algorithm, the variational circuit *V* is optimized to minimize the discrepancy between the energy expectation and the ground state energy, instead of maximizing its overlap with a desired evolution operator. We write the QAOA cost function Equation (7) as

(11) $$C^{\mathrm{QAOA}}\left(s\right)=\left|E_{g}-\left\langle \psi (s\right)|H|\psi (s)\rangle \right|,$$

where the expectation of the Hamiltonian *H* is taken with respect to $|\psi (s)\rangle =V\left(s\right))|\psi_{0}\rangle$, the state evolved from $|\psi_{0}\rangle =|000\ldots \rangle$ under the variationally optimized operator *V*.

From Figure 2, the variational circuit also consists of (non-parametrized) two-qubit entangling gates known as Echoed Cross Resonance (ECR) gates, which are natively supported on the IBM Quantum processors that we use. Functionally equivalent to CX gates, they are defined by

(12) $$ECR=\frac{1}{\sqrt{2}}\left(\begin{array}{cccc} 0 & 1 & 0 & i\\ 1 & 0 & -i & 0\\ 0 & i & 0 & 1\\ -i & 0 & 1 & 0 \end{array}\right).$$

However, in our local noisy simulations, it is more straightforward to include noise effects in CX gates through imperfect X operations. Therefore, we use CX gates for all local simulations in this work, with each ECR gate decomposed as $\left(H⊗I\right)CX\left(I⊗S^{†}\right)CX\left(H⊗X\right)$, with $S^{†}$ denoting the S-adjoint gate.

In our study, the implementation of QITE requires deeper circuits with a larger number of trainable parameters. The 9-qubit instance shown later requires only 2 layers in the QAOA circuit, whereas the corresponding QITE circuit demands between 4 and 8 layers to achieve convergence. Thus, QITE in turn results in a substantially higher classical optimization cost.

In the present implementation, QITE has two main resource bottlenecks. First, it requires deeper variational circuits with more trainable parameters than QAOA. For the nine-qubit portfolio instances considered here, QAOA uses two variational layers, whereas QITE requires four to eight layers to achieve convergence. Second, our QITE construction explicitly forms and compiles an ancilla-extended unitary acting on n+1 qubits. The corresponding matrix has dimension $2^{n+1}$, leading to approximately $O(4^{n})$ memory requirements and $O(8^{n})$ dense-matrix decomposition cost, up to polynomial prefactors. Consequently, the present QITE implementation is restricted to small system sizes by both variational-circuit depth and classical preprocessing cost. This restriction is specific to the explicit dense-unitary construction used in this work and should not be interpreted as a fundamental limitation of all QITE implementations. Moreover, in Appendix E, we discuss quantum annealing as an alternative preparation strategy and highlight the practical challenges associated with implementing this approach on quantum hardware.

## 4. Benchmarking Results

### 4.1. Benchmarking Simulations of QAOA

In this section, we first evaluate the performance of the QAOA method, with the structure of the variational circuits detailed in Figure 2. During the optimization process, the cost function, defined by the energy difference in Equation (7), is minimized to approximate the ground state of the target Hamiltonian.

#### 4.1.1. Noiseless QAOA Simulations

**Figure 3:**
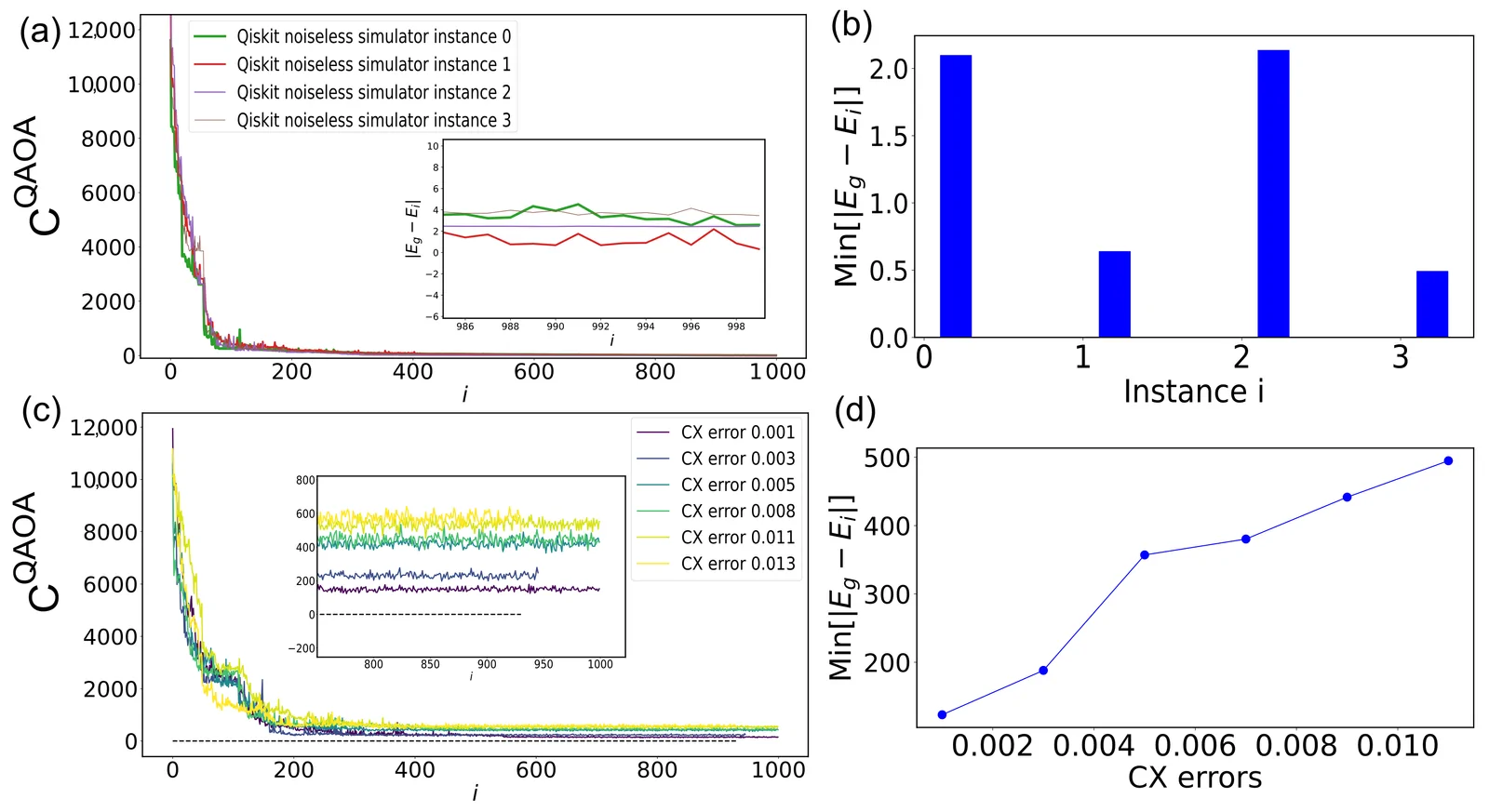
Evaluation of QAOA robustness in estimating the ground-state energy under noiseless and noisy simulations. Here, the model is defined by Equation (5), *i* denotes the number of optimization iterations, and the cost function (Equation (7)) quantifies the deviation between the exact ground-state energy and the estimated energy during the optimization process. (**a**) Convergence of the cost function for four representative problem instances simulated on a noiseless Qiskit backend. All instances demonstrate rapid and consistent convergence. The inset zooms in onto the deviation of $E_{i}$ from the true ground state energy $E_{g}$ at large *i*, with small fluctuations indicative of good convergence. (**b**) Minimum energy deviations (minimum values of the cost function throughout optimization) for each instance from (**a**), all exhibiting the good convergence $C^{\prime \prime }<2.5$. (**c**) Convergence behavior of the QAOA simulations on local noisy simulators under different CX error rates. As noise increases, the optimization becomes unstable and energy estimates deviate significantly from the ground state, as evidenced by the significant oscillations shown in the inset. (**d**) Minimum energy deviations as a function of CX error levels, where each point corresponds to the lowest $|E_{g}-E_{i}|$ in (**c**). As the CX error rates increase, the ground state convergence also deteriorates roughly proportionally. For (**c**,**d**), we select the first instance in (**a**) here. Note that different instances correspond to Hamiltonians with distinct parameters corresponding to different sets of generated asset data, and more details are shown in Appendix B. The system size for the Hamiltonian is L=9 qubits.

As depicted in Figure 3a, we implement the QAOA procedure using a noiseless local simulator. Here, in each optimization iteration, we perform a full round of circuit simulation (distinct from an actual circuit run in Qiskit) and subsequently update the trainable parameters based on the feedback. For these noiseless simulations, the optimization exhibits near-perfect convergence towards $E_{g}$ after approximately 600–1000 iterations. However, this high accuracy comes at the cost of significant quantum resources, requiring over 600 circuit evaluations on quantum hardware. The lowest discrepancy $\mathrm{Min}|E_{g}-E_{i}|$ from the true ground state energy $E_{g}$ achieved throughout the QAOA optimization process in each instance is presented in Figure 3b (see Appendix I for more details). Here, the optimized circuits converge to $C^{\mathrm{QAOA}}\left(s\right)<2.5$, indicating good approximations to the ground state, as further detailed in Appendix F. Note that different problem instances correspond to Hamiltonians with distinct parameter sets derived from independently generated asset data, as detailed in Appendix B. Consequently, their ground states are associated with different optimal bitstrings. That said, even in the absence of noise, the finite number of circuit executions introduces intrinsic sampling errors, preventing convergence to the exact ground state.

#### 4.1.2. QAOA Simulations with Controlled Noise

We then conduct controlled noisy simulations of QAOA on a local simulator, and the effect of CX gate noise on the trajectory of this optimization process is presented in Figure 3c. Under increasing CX noise (error rates), the optimization becomes highly unstable and exhibits no clear convergence, highlighting the significant impact of noise on QAOA performance. This trend is further illustrated in Figure 3d, where it is evident that the minimum energy achieved during optimization increases rapidly with growing CX gate error rates. Even for a 0.001 CX error rate, which is comparable to that in contemporary IBM quantum processors, $E_{g}-E_{i}$ is already far larger than that from noiseless simulations.

In this work, QAOA optimization is performed using the COBYLA optimizer [73], while the corresponding quantum state measurements are collected from a noisy simulator. While COBYLA is designed to handle constrained problems efficiently, it relies on iterative evaluations of the cost function. When those evaluations are affected by hardware noise, the optimizer struggles to navigate the resulting rugged energy landscape, often leading to poor convergence behavior. The controlled local simulations considered here isolate two-qubit gate errors rather than attempting to reproduce every noise process present on a quantum processor. This choice is motivated by the shallow variational circuits used in this work. In particular, the nine-qubit QAOA ansatz contains only two variational layers, which limits the circuit duration and hence the accumulation of relaxation and dephasing. By contrast, entangling operations are required throughout the ansatz and directly generate the correlations needed to represent the optimized portfolio state. Their comparatively large error rates and repeated occurrence, therefore, make two-qubit gate errors the most significant circuit-level noise source for the shallow circuits considered here [see Appendix F for more details]. The resulting model should nevertheless be interpreted as a controlled sensitivity analysis rather than a complete device-level noise model.

#### 4.1.3. Finite-Size QAOA Behavior and Resource Considerations

**Figure 4:**
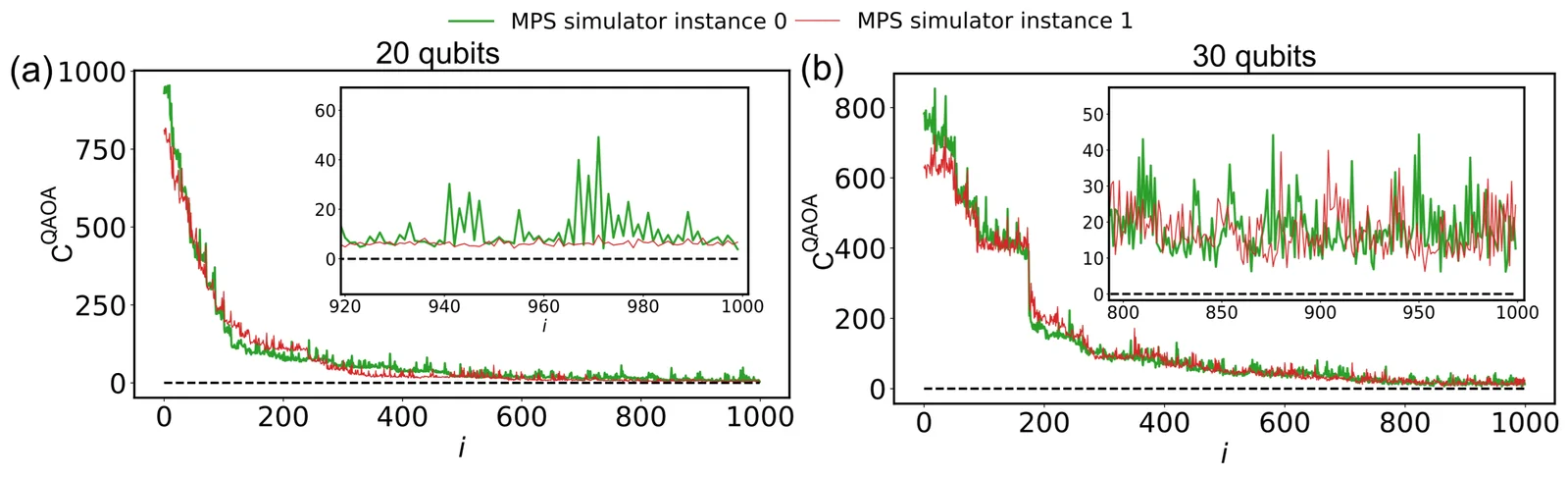
Illustration of the scalability of Quantum Approximate Optimization Algorithm (QAOA) to larger-scale simulations. We evaluate the cost function (Equation (11)) for 20- and 30-qubit problems, as shown in panels (**a**) and (**b**), respectively, using a noiseless matrix product state (MPS) simulator, where *i* denotes the number of optimization iterations. The zoomed-in regions highlight growing instability in the optimization landscape as the system size increases. Despite notable instability at these scales, the cost function ultimately converges to a reasonable energy close to the exact ground-state energy, orders of magnitude lower than that of the initial state.

To examine how the present QAOA implementation behaves beyond the nine-qubit instances, we consider 20- and 30-qubit portfolio Hamiltonians using a noiseless matrix-product-state simulator [74]. As shown in Figure 4, the optimization substantially reduces the cost function for both system sizes. The 20-qubit instances exhibit relatively stable convergence, whereas the 30-qubit results display larger fluctuations during the later optimization stages. These results show that the shallow variational ansatz remains capable of lowering the energy for the intermediate system sizes considered here. They should not, however, be interpreted as evidence of asymptotic or hardware-level scalability, because the analysis is restricted to a small number of noiseless instances and does not establish how the convergence rate or required circuit depth behaves for general large-scale portfolio problems [see Appendix H for more details].

The resource growth can nevertheless be characterized from the circuit structure. For *n* qubits and *d* variational layers, the ansatz in Figure 2 contains 3nd trainable parameters and approximately d(n−1) nearest-neighbor two-qubit gates before transpilation. The logical circuit depth grows as O(d) when non-overlapping entangling gates are executed in parallel, although limited hardware connectivity can introduce additional routing gates. If the optimizer requires $N_{\mathrm{eval}}$ evaluations and each energy estimate uses $N_{s}$ shots, the QAOA sampling workload is $N_{\mathrm{eval}}N_{s}$ circuit executions. All $Z_{i}$ and $Z_{i}Z_{j}$ contributions to the portfolio Hamiltonian commute and can, therefore, be extracted from the same computational-basis measurements, avoiding a separate measurement circuit for every Hamiltonian term. Nevertheless, the number of pairwise covariance contributions is generally $O(n^{2})$, and the required number of optimization evaluations remains problem-, optimizer-, and noise-dependent.

The data in Figure 4 are, therefore, used only to characterize finite-size convergence within the studied numerical setting. In particular, the increased fluctuations observed for 30 qubits indicate that increasing the number of available qubits does not by itself guarantee stable optimization.

### 4.2. Comparison Between QAOA and QITE

#### 4.2.1. Benchmarking from Predicted Returns

**Figure 5:**
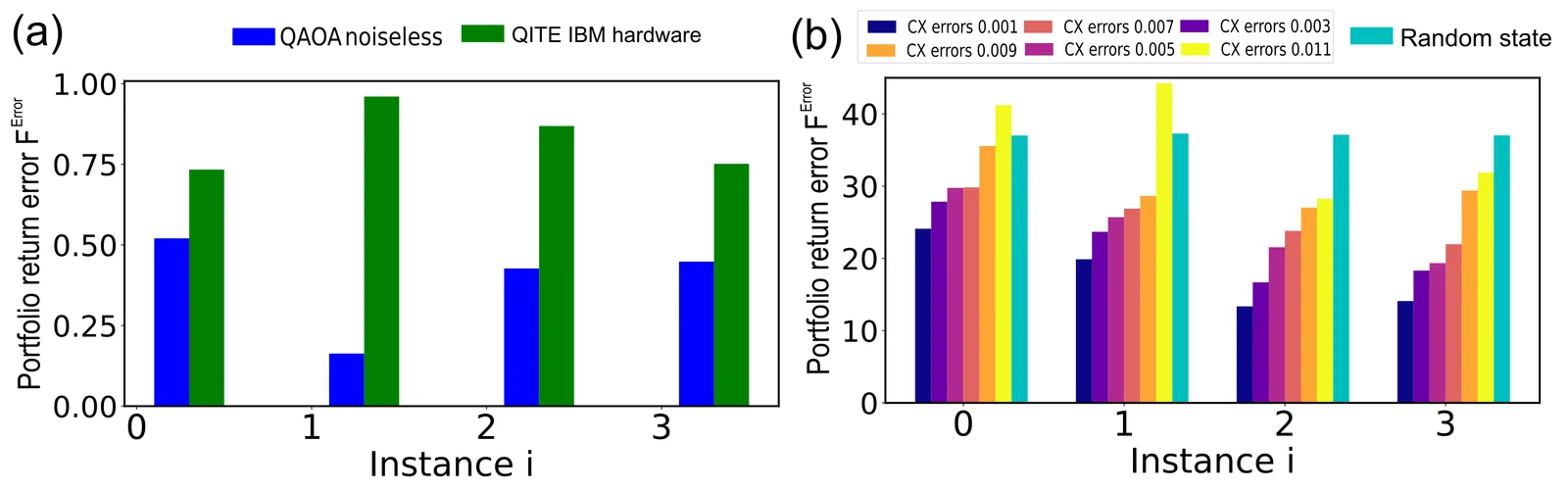
Assessment of QAOA and QITE performance via return errors $F^{\mathrm{Error}}=F^{\mathrm{ideal}}-F^{\mathrm{circuit}}$ (Equation (13)). (**a**) Return errors for four selected instances obtained from noiseless QAOA simulations (blue) and QITE executed on IBM Quantum hardware (green) with typical 2-qubit gate error rate of 0.007. Both methods yield results that closely approximate the exact returns, with QAOA achieving slightly better accuracy with the benefit of ideal noiseless conditions. (**b**) Return errors from noisy QAOA simulations on a local simulator with a range of two-qubit (CX) gate error rates representative of those in cutting-edge digital quantum processors. Higher noise levels lead to an increase in errors across all instances, highlighting the sensitivity of QAOA to noise. Cyan bars denote outcomes computed under random states, which represent return errors for completely unoptimized reference cases. Error bars represent standard deviations computed over 10 random samples. The selected instances are the same as those used in Figure 3.

**Figure A1:**
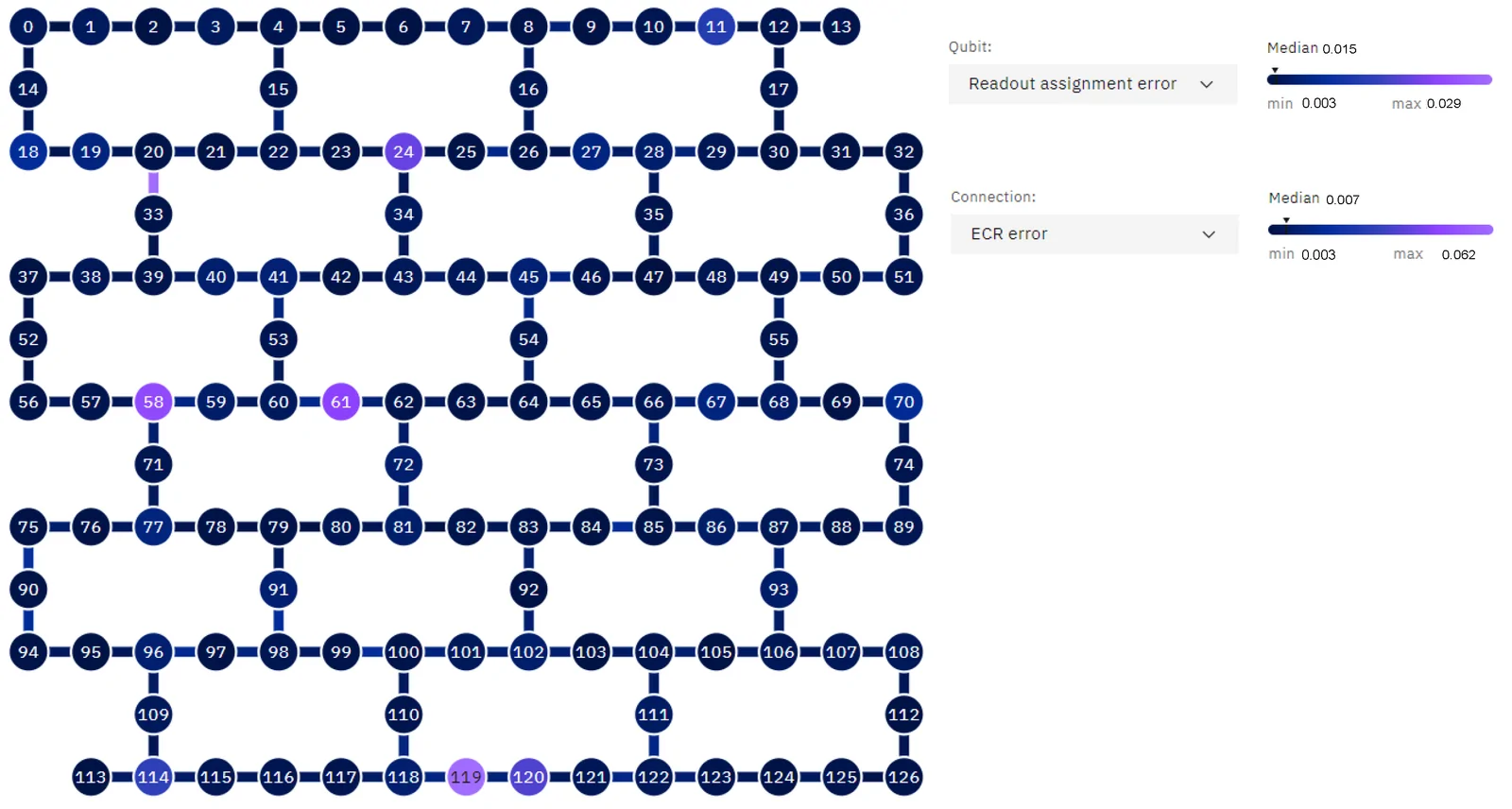
The error map of the IBM “Brisbane” device. For simulations in our work, we select the qubit chain [0,1,2,3,4,5,6,7,8,9,10,11,12], with the mean ECR error rate around 0.007.

In this section, we compare the performance of QAOA and QITE in terms of their predicted returns *F*, as defined and computed by Equation (1). Using the same representative instances, we compute the return error between the circuit outputs and the exact (ideal) results, defined as

(13) $$F^{\mathrm{Error}}=F^{\mathrm{ideal}}-F^{\mathrm{circuit}},$$

where $F^{\mathrm{ideal}}\approx 0$, and $F^{\mathrm{circuit}}<0$ denotes loss in portfolio value due to suboptimal outcomes. Figure 5 illustrates the simulated results of such returns. Figure 5a demonstrates that, remarkably, the QITE algorithm executed on IBM quantum hardware (with ≈0.007 ECR/CX error rate, as obtained from Figure A1 from Appendix C) predicts return values that exhibit very low $F^{\mathrm{Error}}<1$, almost comparable to that from the QAOA instances obtained in ideal (noiseless) settings. A direct hardware implementation of the complete QAOA optimization loop was not performed because each parameter update depends sequentially on the measurement result from the preceding iteration. The nine-qubit optimizations considered here require approximately 600–1000 cost-function evaluations, each involving circuit execution, measurement, classical processing, and submission of an updated circuit. Although runtime sessions can reduce repeated queue delays, the complete loop still incurs substantial communication latency, QPU usage, and wall-clock cost on a shared-access quantum processor. Extending this procedure to multiple problem instances and repeated optimization runs would, therefore, require a hardware allocation beyond the scope of the present study. We consequently evaluate QAOA using local simulators with controlled two-qubit error rates, while the computationally demanding QITE compilation is performed offline and only the resulting fixed circuit is executed on IBM hardware.

**Figure 6:**
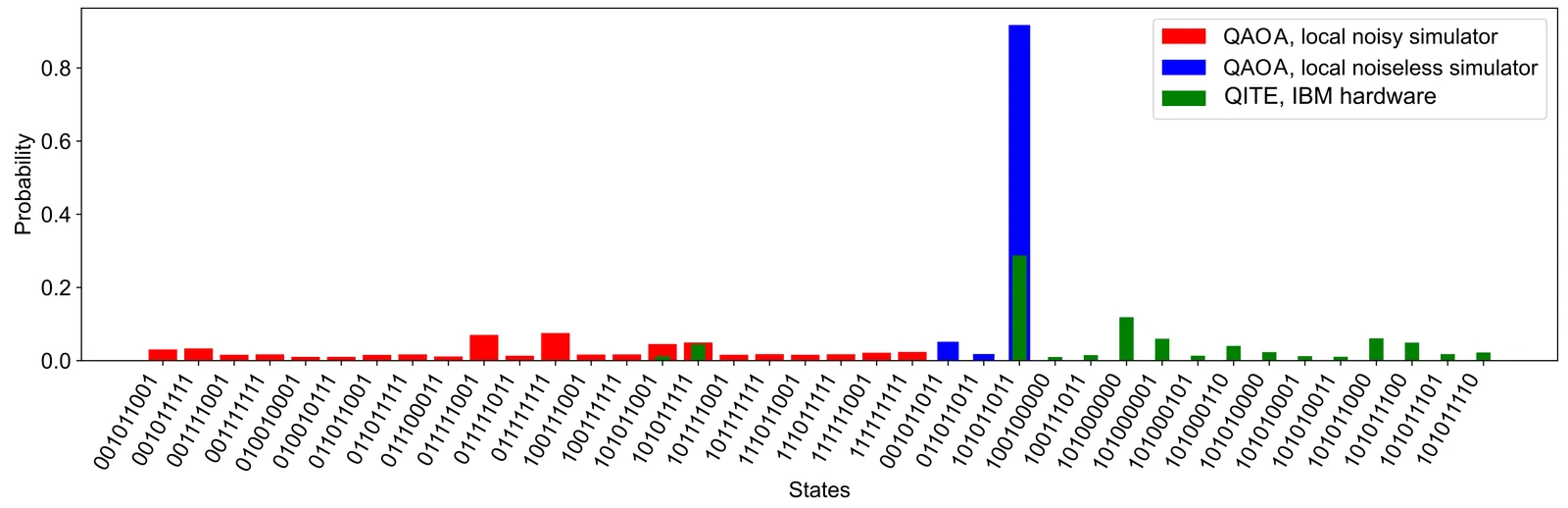
Probability distribution of the optimal states obtained from QAOA and QITE for the representative instance 0 (Figure 3), so as to compare their convergence towards the ideal bitstring 101011011 (instance 0 in Figure 3). Results are shown in green, blue, and red, corresponding to IBM Quantum simulations of the QITE method, noiseless QAOA simulations, and noisy QAOA simulations (local simulator with realistic noise levels of 0.007 (two-qubit gates), Appendix C), respectively. In simulations on IBM hardware, prominent peaks (green) successfully identify the optimal state. For the noiseless QAOA simulations (blue), the distribution exhibits an even more distinct sharp peak at the optimal solution bitstring. In the noisy QAOA simulation (red), noise effects significantly impact performance, preventing the identification of the optimal state.

Because the two algorithms were not evaluated using matched hardware workflows, Figure 6 should not be interpreted as establishing an intrinsic hardware advantage of QITE over QAOA. The hardware results demonstrate that the compiled QITE circuit retains an identifiable optimal solution under the device conditions considered here. A direct algorithmic comparison would require QAOA and QITE to be executed on the same processor with matched qubit layouts, shot budgets, transpilation settings, mitigation procedures, and total computational resources.

To put the excellent performance of the QITE hardware runs into perspective, we compare the return error $F^{\mathrm{Error}}$ from QAOA with simulated noise of similar magnitude Figure 5b. Saliently, even the smallest levels of noise significantly affect QAOA performance, with return errors exceeding $F^{\mathrm{Error}}>20$, and increasing further as noise levels rise. Notably, the optimal results obtained from QAOA are not stable: under a moderately elevated noise level (0.011), the first two instances exhibit a sharp increase in return error. This instability arises because noise can introduce temporal fluctuations in the measured cost function, leading to a non-smooth landscape in optimization. As a result, the performance of QAOA becomes highly sensitive to both the specific problem instance and the stochastic behavior of the circuit runs.

To further contextualize our $F^{\mathrm{Error}}$ results, we also compute returns from reference states with uncorrelated uniformly random coefficients, which represent completely unoptimized outcomes (These random states are not produced by random circuits; instead, we generate them numerically as $|\psi \rangle =\sum_{i}a_{i}|\psi_{i}\rangle /\left|\right|\sum_{i}a_{i}|\psi_{i}\rangle \left|\right|$, which are normalized. Here, the coefficients $a_{i}$ are randomly sampled from a uniform distribution within [0,1], and $|\psi_{i}\rangle$ range over the computational basis states.). As shown in Figure 5b (cyan bars), these unoptimized reference random states exhibit large errors of $F^{\mathrm{Error}}\approx 38$, which substantially exceed those in Figure 5a, and are also larger than the low-noise QAOA results also plotted in Figure 5b. However, under significant but realistic noise levels, such as a CX error rate of 0.011, QAOA performance error approaches the level of these fully unoptimized outcomes, offering no optimization value at all (see Appendix G for more details).

#### 4.2.2. Benchmarking from Measured Optimal State Distribution

We now examine the actual solution states obtained from both QITE and QAOA, which represent the ideal portfolio composition. In principle, the ideal optimal solution corresponds to a single bitstring with the highest return. Thus, to assess how the outputs of QAOA and QITE deviate from the ideal solution, we examine a representative problem instance and analyze the full probability distributions of measured quantum states.

As shown in Figure 6, the QITE results (green bars) obtained from simulations on IBM hardware exhibit a reasonably prominent peak at the bitstring 101011011 corresponding to the exact optimal solution. Although the obtained state probability distribution is broadened due to the presence of noise, the optimal bitstring remains clearly identifiable. For comparison, the blue bars in Figure 6 display the probability distribution obtained from the noiseless QAOA simulation with the same instance. In this ideal noiseless setting, QAOA yields a nearly perfectly localized state on this optimal bitstring (see Appendix F). In contrast, QAOA results (red bars) conducted on a local simulator under realistic errors (see Appendix C) fail to reveal any distinct peak corresponding to the optimal solution. This behavior highlights the instability under noise, consistent with the unstable convergence trends observed in Figure 3.

**Figure 7:**
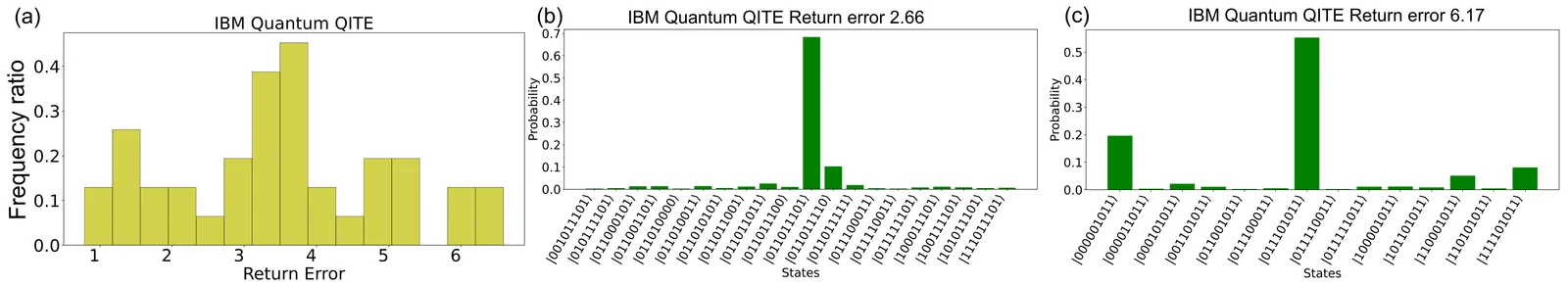
Further performance assessment of the Quantum Imaginary-Time Evolution (QITE) algorithm in noisy quantum processors. We perform additional simulations for 50 problem instances to evaluate the robustness of QITE in small-scale (9 qubits) settings. (**a**) Yellow bars denote the histogram of return errors $F^{\mathrm{Error}}$ (Equation (13)) for simulations conducted on the IBM Quantum processor, spanning relatively small values of 1to6. (**b**,**c**) State probability distributions for two selected instances from (**a**), with corresponding return errors of 2.66 and 6.17, respectively. In both cases, the optimal strings remain clearly identifiable despite the non-negligible return errors. The Hamiltonian corresponds to a system size of L=9 qubits. Note that these 50 instances represent different Hamiltonians, and more details are shown in Appendix B.

To further validate the robustness of QITE, we simulate the QITE circuit for an additional 50 portfolio optimization instances on the IBM quantum computer, also with L=9 qubits. These simulations are performed on both IBM Quantum hardware. As shown in Figure 7, the return errors $F^{\mathrm{Error}}$ range from about 1 to 6, which is much smaller than those from QAOA with a similar simulated CX gate error rate of 0.007 (see Appendix C), as previously shown in Figure 5. Note that the return error range can arise from variations in the optimization quality of circuits across different instances, as well as from noise fluctuations in simulations. Figure 7b,c present the measured solution state distributions for two representative instances drawn from Figure 7a. Despite higher return errors of 2.66 and 6.17, the optimal bitstring still remains prominent.

## 5. Conclusions

In this work, we benchmarked two quantum algorithms, QAOA and QITE, which are widely used in digital quantum simulations for solving not just the Markowitz portfolio optimization problem, but also other diverse problems with quantum Ising formulations [75,76,77,78]. Our results show that the QAOA method achieves excellent convergence to the exact ground state in noiseless simulations when hardware noise is negligible or extensively Under the controlled noise model considered here, QAOA is particularly sensitive to two-qubit gate errors entering its repeated quantum–classical optimization loop. These errors modify the measured cost function at every iteration and can destabilize the subsequent parameter updates even though the variational circuit itself is shallow. The QITE circuits retain identifiable optimal portfolio states in the nine-qubit IBM hardware experiments, which include the aggregate effects of gate errors, readout errors, decoherence, calibration drift, and other device-dependent imperfections. These results demonstrate favorable finite-size robustness under the hardware conditions studied here, rather than a universal noise advantage of QITE over QAOA.

From a resource perspective, as touched on earlier in Section 3.3, QAOA demands extensive quantum resources, typically requiring hundreds to thousands of circuit executions, but relatively low classical computational cost. However, QITE, on the other hand, requires less quantum cost but substantial classical computational cost due to the need for extensive circuit training. As illustrated in Figure 3 and Figure 4, the 9-qubit instance of QAOA requires a few hundred iterations for reasonable convergence, which increases to around 1000 iterations for the 20-qubit case. For QITE, only a single round of circuit simulation is required, equivalent to the cost of one QAOA iteration, resulting in markedly lower quantum resource requirements. For problems of the same scale, QAOA requires hundreds of times more quantum resources than QITE.

On quantum hardware, qubit measurements are straightforward and can be performed on systems with over 100 qubits. As a result, in terms of scalability, QAOA holds greater theoretical promise for tackling larger problem instances, if hardware noise can be effectively mitigated. A detailed side-by-side comparison of both methods is summarized in Table 1.

The distinction between hardware architectures is also relevant for future scaling. Our experiments use a superconducting quantum processor, whose restricted connectivity can require additional routing operations when implementing densely coupled optimization problems. Trapped-ion processors provide high or effectively all-to-all connectivity and are, therefore, particularly advantageous for such dense interactions. Scaling the present framework is not restricted exclusively to trapped-ion devices, but its implementation on larger superconducting processors will require efficient routing, problem decomposition, or connectivity-aware ansatz design.

Overall, our comparative analysis of the noise resilience and scalability in quantum optimization strategies suggests that the choice between QAOA and QITE should be guided by the specific noise characteristics of the quantum device, the availability of classical computational resources, and the complexity and scale of the optimization task [37,72,79,80,81,82,83].

## Figures and Tables

**Table 1:** Comparison of the QAOA and QITE implementations studied in this work. QAOA is evaluated through noiseless and controlled-noise simulations, whereas the offline-compiled QITE circuits are executed on IBM hardware. The comparison, therefore, does not constitute a matched hardware benchmark.

Property	QAOA	QITE
Evidence presented	Noiseless and controlled-noise simulations for 9-qubit instances, together with noiseless MPS simulations for 20 and 30 qubits.	IBM hardware experiments for 9 system qubits and one ancilla, including four representative and 50 additional instances.
Observed performance	Accurate convergence in the 9-qubit noiseless simulations and substantial energy reduction at 20 and 30 qubits. Increasing two-qubit noise destabilizes the iterative optimization.	The optimal bitstring remains identifiable under the hardware conditions studied, although the measured distributions are broadened by noise.
Circuit and workflow	Uses two variational layers for the 9-qubit instances and requires approximately 600–1000 sequential quantum–classical cost evaluations.	Uses four to eight variational layers. The circuit is compiled offline, after which only the fixed circuit is executed with ancilla post-selection.
Dominant cost	Large online sampling and feedback cost. With $10^{4}$ shots per evaluation, one 9-qubit instance requires approximately $6\times 10^{6}$–$10^{7}$ circuit executions.	Large offline classical cost from constructing and compiling the ancilla-extended unitary, together with deeper circuits and post-selection overhead.
Scope of scaling	The 20- and 30-qubit results provide finite-size evidence but do not establish asymptotic or noisy-hardware scalability.	The present dense-unitary construction has approximately $O(4^{n})$ memory and $O(8^{n})$ decomposition costs, which restrict it to small systems.

**Figure A2:**
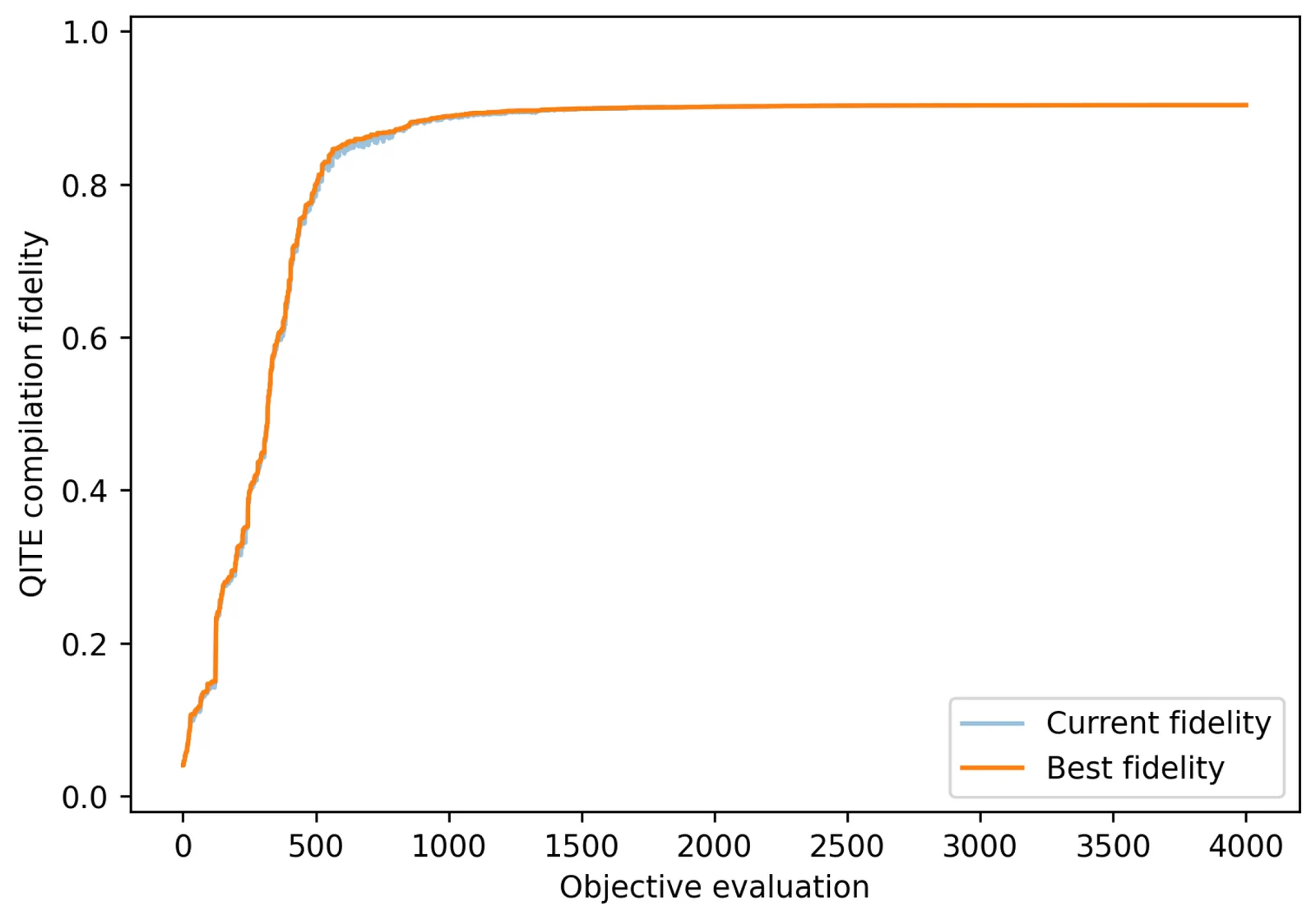
Convergence of the QITE variational compilation for the representative portfolio instance used in the main text. The circuit contains four variational layers and is optimized by maximizing the compilation fidelity $F_{\mathrm{comp}}={\left|\langle \psi_{\mathrm{target}}|\psi_{\mathrm{circuit}}\left(s\right)\rangle \right|}^{2}$. The light-blue curve shows the fidelity obtained at each objective-function evaluation, while the orange curve denotes the highest fidelity reached up to that evaluation. The fidelity increases rapidly during the first approximately 600 evaluations and subsequently approaches a plateau near $F_{\mathrm{comp}}≃0.90$. The system consists of 10 qubits, and the circuit is built by 4 layers.

**Figure A3:**
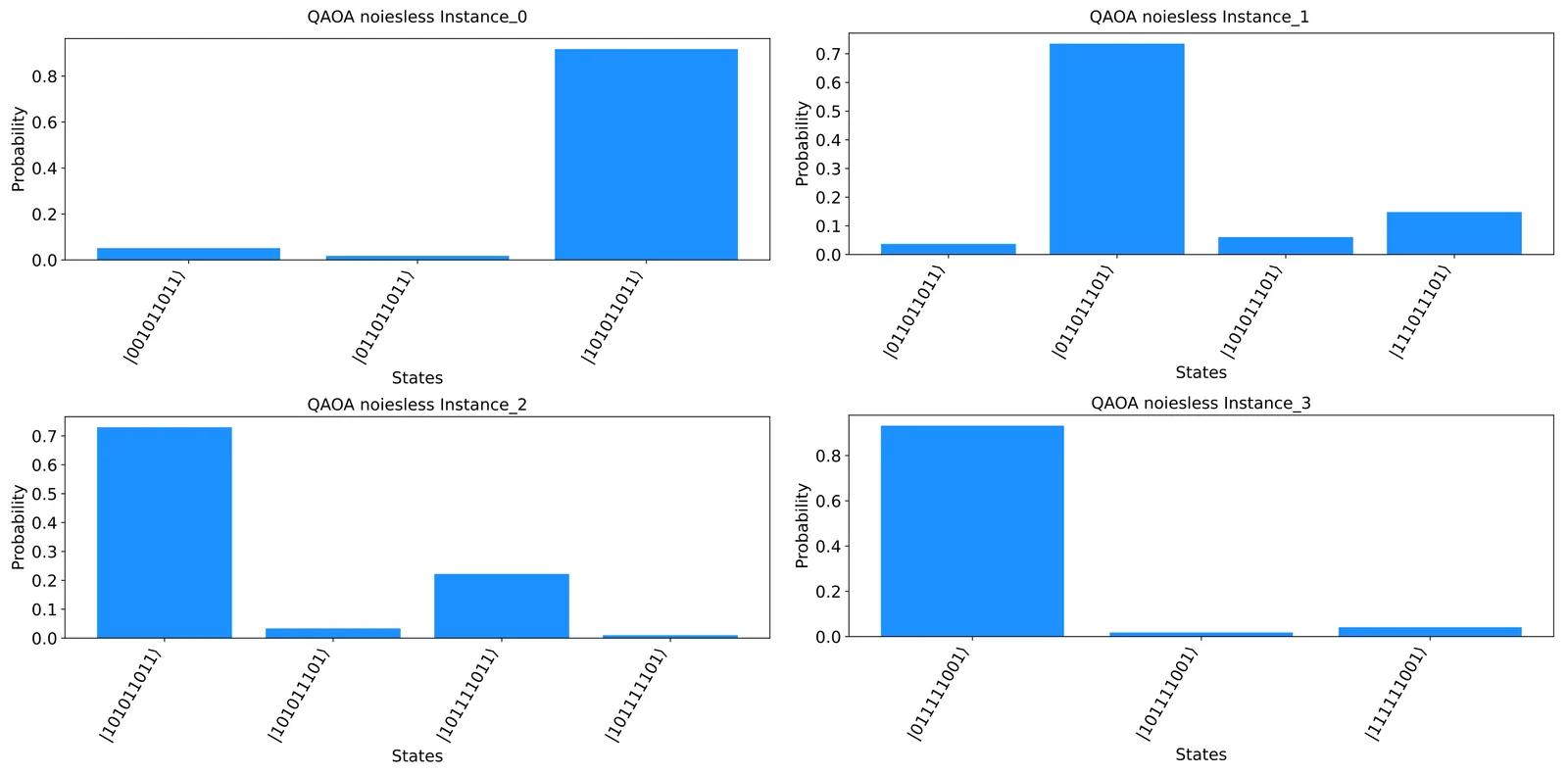
Probability distributions from noiseless QAOA simulations for four representative problem instances (Figure 5, main text). In all cases, a distinct peak appears at the optimal bitstring, indicating successful convergence.

**Figure A4:**
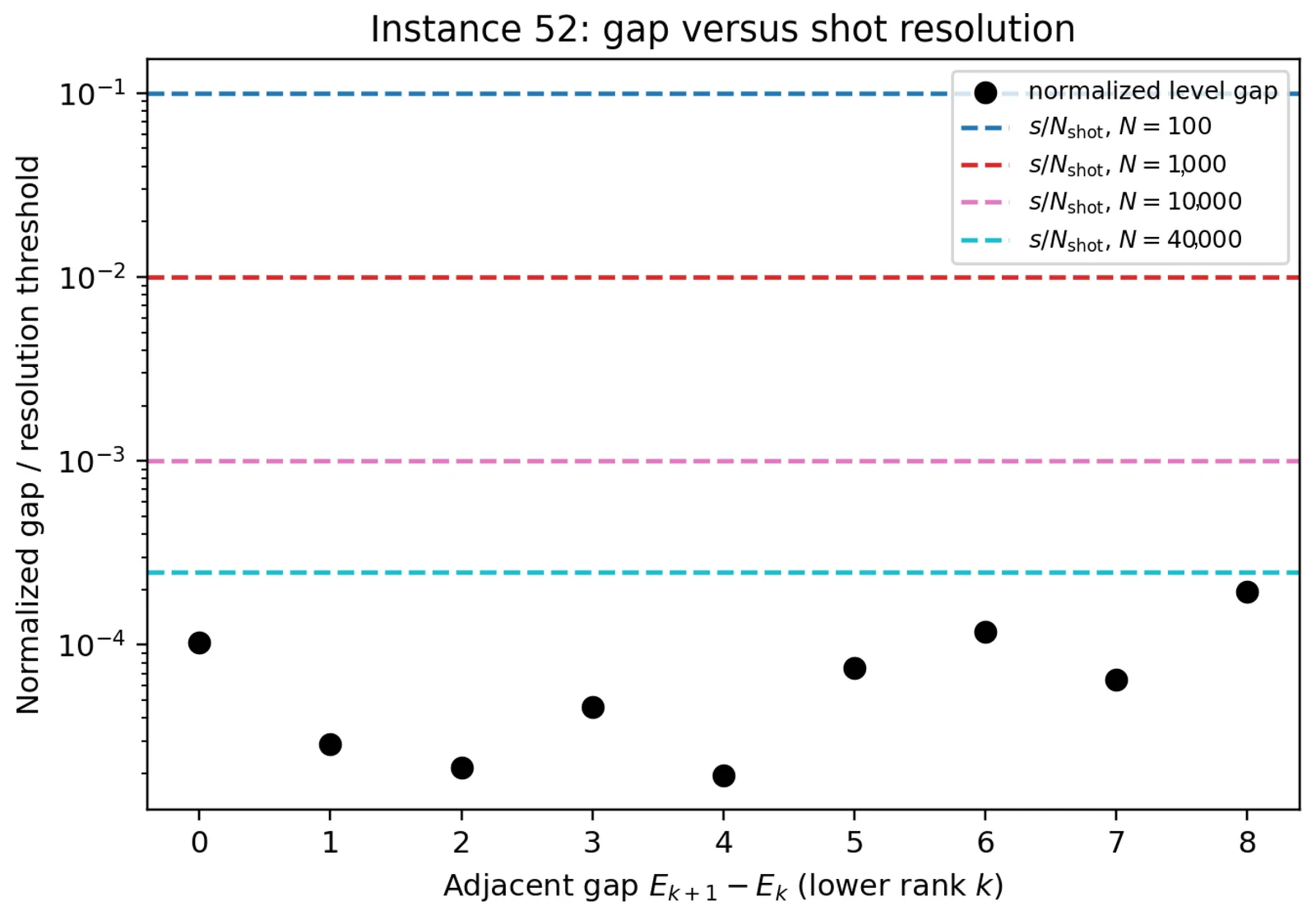
Shot-resolution analysis for the low-energy spectrum of portfolio instance 52. Black circles show the normalized adjacent energy gaps ${\tilde{\Delta }}_{k}=\left(E_{k+1}-E_{k}\right)/\left(E_{\max}-E_{\min}\right)$ between the first ten distinct energy levels. Dashed lines indicate the resolution threshold $s/N_{\mathrm{shot}}$ for $N_{\mathrm{shot}}=$100, 1000, 10,000, and 40,000, with safety factor s=10. A gap is regarded as resolved when ${\tilde{\Delta }}_{k}\geq s/N_{\mathrm{shot}}$. For this instance, all displayed gaps remain below the $N_{\mathrm{shot}}$ = 40,000 threshold, indicating that a larger shot number is required to distinguish the neighboring low-energy levels according to this criterion. The system consists of 9 qubits.

## Data Availability

The data presented in this study are available at https://zenodo.org/records/16788658 (accessed on 5 August 2026). More data are available on request from the corresponding author.

## Appendix A. Correspondence Between the Markowitz Portfolio Model and the Ising Hamiltonian

In this section, we detail the construction of the Ising Hamiltonian for the Markowitz portfolio selection problem described by Equation (1). We begin by reformulating the total expected return using binary variables, a necessary step for quantum computing applications [43].

We use $z_{u}$ to represent the investment in asset *u*, quantified as an integer number of quantized minimum investment fractions. We can express each integer $z_{u}$ using a standard binary representation with *w* binary variables, $x_{i(u,k)}\in \left\{0,1\right\}$:

(A1) $$z_{u}=\sum_{k=1}^{w}2^{k-1}x_{i(u,k)},$$

where i(u,k)=(u−1)w+k,k=1,2,…,w. In this expression, the variables $\left\{x_{i(u,1)},\ldots ,x_{i(u,w)}\right\}$ are the binary digits of $z_{u}$. For example, if we use w=3 bits to represent the investment in the u-th asset, an amount of $z_{u}=5$ (which is $101_{2}$ in binary) would be encoded by setting $x_{i(u,1)}=1$, $x_{i(u,2)}=0$, and $x_{i(u,3)}=1$, since $5=\left(1\cdot 2^{0}\right)+\left(0\cdot 2^{1}\right)+\left(1\cdot 2^{2}\right)$. Substituting this binary expansion of $z_{u}$ (Equation (A1)) into the formula for total expected return in Equation (1) yields:

(A2) $$\sum_{u=1}^{m}r_{u}z_{u}=\sum_{u=1}^{m}r_{u}\left(\sum_{k=1}^{w}2^{k-1}x_{i(u,k)}\right)=\sum_{u=1}^{m}\sum_{k=1}^{w}2^{k-1}r_{u}x_{i(u,k)}.$$

Here, *m* is the total number of assets, *w* is the number of binary variables (or “qubits”) allocated to each asset, and $r_{u}$ is the expected return for the *u*-th asset. The function i(u,k)=(u−1)w+k simply assigns a unique index to each binary variable across all assets.

The corresponding covariance term is rewritten as follows

(A3) $$-\sum_{u,v}^{m}c_{u,v}z_{u}z_{v}=-\sum_{u,v}^{m}\sum_{k=1}^{w}\sum_{k^{\prime }=1}^{w}2^{k-1}2^{k^{\prime }-1}x_{i(u,k)}x_{j(v,k^{\prime })}.$$

The allocation constraint is given by the following:

(A4) $$-(\sum_{u=1}^{m}p_{w}bz_{u}-b{)}^{2}=-(\sum_{u=1}^{m}\sum_{k=1}^{w}2^{k-1}p_{w}bx_{i(u,k)}-b{)}^{2},$$

where *b* is the budget and $p_{w}=1/2^{w-1}$ is the quantized minimum investment fraction.

Based on such a binary representation, Equation (1) from the main text can be rewritten as follows:

(A5) $$\underset{z}{max}\left[\theta_{1}\sum_{i=1}^{n}r_{i}x_{i}-\theta_{2}\sum_{i,j=1}^{n}c_{i,j}x_{i}x_{j}-\theta_{3}(\sum_{i=1}^{n}2^{k-1}bp_{w}x_{i}-b{)}^{2}\right],$$

with the following rescaling parameters:

(A6) $$\begin{array}{cc} n & =mw,\\ r_{i} & =2^{k-1}r_{u},\\ c_{i,j} & =2^{k-1}2^{k^{\prime }-1}c_{u,v}. \end{array}$$

The above optimization is equivalent to a Quadratic Unconstrained Binary Optimization (QUBO) problem, expressed as follows:

(A7) $$\underset{x}{min}\left(\sum_{i}^{n}q_{i}x_{i}+\sum_{i,j}^{n}Q_{i,j}x_{i}x_{j}+\gamma \right),$$

where the parameters are defined as follows:

(A8) $$\begin{array}{cc} q_{i} & =-\theta_{1}r_{i}-2\theta_{3}b^{2}p_{w}2^{k-1},\\ Q_{i,j} & =\theta_{2}c_{i,j}+\theta_{3}b^{2}p_{w}^{2}2^{k-1}2^{k^{\prime }-1},\\ \gamma & =\theta_{3}b^{2}. \end{array}$$

By transforming $x_{i}=\frac{1}{2}\left(1+\sigma_{i}^{z}\right)$, the above optimization can be reduced to finding the ground state of the following Ising Hamiltonian:

(A9) $$H=\sum_{i}h_{i}\sigma_{i}^{z}+\sum_{i,j}J_{i,j}\sigma_{i}^{z}\sigma_{j}^{z}+\delta ,$$

with the corresponding parameters:

(A10) $$\begin{array}{cc} J_{i,j} & =\frac{1}{4}Q_{i,j},\\ h_{i} & =\frac{1}{2}q_{i}+\frac{1}{2}\sum_{j}Q_{i,j},\\ \delta & =\frac{1}{4}\sum_{i,j}Q_{i,j}+\frac{1}{2}\sum_{i}^{n}q_{i}+\gamma . \end{array}$$

In our implementation, we discard the constant term δ, as it does not affect the optimization process.

The resulting interaction matrix is formally dense rather than restricted to nearest-neighbor couplings. However, the dense structure is highly constrained. For the instances used in this work, the data-dependent inter-asset long-range contribution is below approximately 0.4% of the total coupling norm, while the dominant long-range component arises from the global budget constraint and has a low-rank collective form. The present Hamiltonians should, therefore, be regarded as structured dense portfolio QUBOs, rather than generic fully connected spin-glass models with strong random frustration. Their optimization difficulty mainly originates from the discrete allocation variables, the competition between return and risk, and the global budget constraint.

## Appendix B. Dataset Preparation

In this section, we outline the method used to prepare our dataset, which simulates the historical prices for financial assets. The asset price dataset is structured as a matrix of dimensions $\left(N_{f},m\right)$, where $N_{f}$ represents the number of historical price points per asset, and *m* is the number of assets under consideration. Each column in this matrix corresponds to the price history of a distinct asset.

The dataset preparation process begins by setting the price generator $a_{u,0}$, for the *u*-th asset. This price generator is randomly selected from the range [b/10,b], where *b* is the total budget. To generate Brownian-like temporal price fluctuations, each subsequent historical price point, $a_{u,l}$, is then generated within a fluctuation range of 75% to 125% of the price generator, specifically, $a_{u,l}=\left(1+\alpha \right)a_{u,0}$. This procedure generates the *u*-th column of the price data matrix. To construct the full matrix, this process is repeated across all assets. Based on the generated price data, the expected return $r_{u}$ and covariance matrix $c_{u,v}$ are calculated using Equation (2) and Equation (3), respectively.

With the calculated price data, we proceed to generate the Ising Hamiltonian problem instances. This is achieved by selecting one combination of the parameters $\theta_{1},\theta_{2},\theta_{3}$ subject to the constraint $\sum_{i}\theta_{i}=1$, where different choices of θ correspond to distinct investment preferences. These parameters, which represent different investor preferences, are integrated with the calculated $r_{u}$ and $c_{u,v}$. Subsequently, the rescaling process described in Equation (A6) is applied. A detailed workflow (9-qubit instance in the main text) is demonstrated as follows, and the corresponding code and data are available at [84]:

1. **Input parameters:** The number of assets is m=3, with each asset having $N_{f}=100$ price points. Each asset is represented using w=3 binary bits (slices), with $p_{w}=1/2^{w-1}$. A total of 100 problem instances are generated, with a total budget b=10. Additional parameters are set to $\theta_{1}=0.8$, $\theta_{2}=0.1$, and $\theta_{3}=0.1$.
2. **Price generation:** For each asset *u*, randomly initialize the price generator $a_{u,0}$ within the range [b/10,b]. Generate historical prices using $a_{u,l}=\left(1+\alpha \right)a_{u,0}$, where α is a random variable uniformly sampled from [−0.25,0.25].
3. **Covariance matrix and expected return:** For each instance:
  - Compute the covariance matrix $c_{uv}$ as defined in Equation (3) of the main text:
    $c_{u,v}=\frac{b^{2}p_{w}^{2}}{a_{u,N_{f}}a_{v,N_{f}}}\cdot \frac{\sum_{l=1}^{N_{f}}\left(a_{u,l}-{\bar{a}}_{u}\right)\left(a_{v,l}-{\bar{a}}_{v}\right)}{N_{f}-1},$ where $\bar{a}$ denotes the mean price of the assets and $p_{w}=2^{1-w}$.
  - Compute daily returns $\Delta a_{u,l}=a_{u,l+1}-a_{u,l}$ and the expected return $r_{u}$ from Equation (2) of the main text: $r_{u}=\frac{bp_{w}}{a_{u,N_{f}}}\cdot \sum_{l=1}^{N_{f}}\frac{a_{u,l+1}-a_{u,l}}{N_{f}-1}.$
4. **Rescaling:** Rescale $c_{uv}$ and $r_{u}$ to $Q_{i,j}$ and $q_{i}$ (parameters for QUBO problems) according to Equations (A6) and (A8).
5. **Ising coupling and field parameters:** The total number of binary variables is m·w=9. For binary indices i,j∈{1,…,9}, the corresponding Ising coupling $J_{ij}$ and Ising field $h_{i}$ are computed based on Equation (A10): $J_{i,j}=\frac{1}{4}Q_{i,j},h_{i}=\frac{1}{2}q_{i}+\frac{1}{2}\sum_{j}Q_{i,j}$.

In this work, for the 9-qubit problem, we generate 100 distinct instances, each associated with a specific Hamiltonian. Consequently, the optimization landscape varies across instances, and the optimal solution bitstring is not universally fixed.

## Appendix C. IBM Quantum Device Used

For simulations in this work, we implement jobs on the IBM “Brisbane” device, and details of the gate errors are shown in Figure A1. Here, we select the qubit chain [0,1,2,3,4,5,6,7,8,9,10,11,12] to be used for our computations. Note that simulations shown in Figure 6 are based on a local noise model calibrated using the error parameters extracted from the “Brisbane“ device [85].

### Scope of the Controlled Noise Model

Real quantum processors are affected by several noise mechanisms, including single- and two-qubit gate errors, state-preparation and measurement errors, relaxation, dephasing, leakage, crosstalk, and calibration drift. The controlled local simulations in the main text isolate two-qubit gate errors and are not intended to reproduce the complete noise environment of the IBM processor. This choice is particularly relevant for the shallow variational circuits considered here. The nine-qubit QAOA ansatz contains two variational layers, so the total circuit duration is relatively short and the accumulation of relaxation and dephasing is correspondingly limited. Entangling gates, however, occur in every variational layer and are necessary for generating the multi-qubit correlations that encode the optimized portfolio state. The selected IBM Brisbane qubit chain has a mean ECR error rate of approximately 0.007, as shown in Figure A1. Two-qubit gate errors, therefore, provide the most significant circuit-level noise contribution for the present shallow implementation and constitute a physically motivated starting point for a controlled noise-sensitivity analysis.

If a circuit contains $N_{2q}$ entangling gates with a characteristic two-qubit error probability $\epsilon_{2q}$, the probability of completing all entangling operations without such an error may be estimated under an independent-error approximation as

(A11) $$P_{\mathrm{no-error}}≃{\left(1-\epsilon_{2q}\right)}^{N_{2q}}≃\exp\left(-N_{2q}\epsilon_{2q}\right).$$

This estimate is not a complete hardware model because realistic errors may be coherent, correlated, time-dependent, and qubit-dependent. Nevertheless, it illustrates why imperfect entangling gates can noticeably affect the output even when the variational circuit itself is shallow.

The effect is particularly important for QAOA because noisy circuit measurements are repeatedly supplied to the classical optimizer. A noisy cost-function estimate can be represented schematically as

(A12) $${\tilde{C}}^{\mathrm{QAOA}}\left(s\right)=C^{\mathrm{QAOA}}\left(s\right)+\delta C_{\mathrm{gate}}\left(s\right)+\delta C_{\mathrm{readout}}\left(s\right)+\delta C_{\mathrm{shot}}\left(s\right),$$

where the correction terms denote gate-induced bias, readout bias, and finite-sampling fluctuations. These contributions affect not only the final measured state but also the optimization trajectory because each noisy cost estimate influences subsequent parameter updates.

The QITE results reported in the main text were obtained directly on IBM quantum hardware and, therefore, include the aggregate effects of gate errors, readout errors, decoherence, routing imperfections, calibration drift, and device-dependent crosstalk. The persistence of identifiable optimal bitstrings demonstrates that the compiled QITE circuits remain effective under the combined hardware noise encountered in the nine-qubit experiments. Because the individual noise channels were not independently varied, however, these results should not be interpreted as establishing universal QITE robustness for arbitrary devices, circuit depths, or system sizes.

## Appendix D. Quantum Imaginary-Time Evolution

In this section, we describe our approach for simulating the non-unitary evolution operator $U_{\mathrm{non}}=e^{-\beta H}$. Since quantum circuits are inherently unitary, this non-unitary operation must be embedded into a larger unitary operator that can be physically implemented. This embedding can be constructed in the following form:

(A13) $$U=\left[\begin{array}{cc} uU_{\mathrm{non}} & B\\ C & D \end{array}\right].$$

where the scaling factor $u^{-2}$ denotes the maximum eigenvalue of $U_{\mathrm{non}}^{†}U_{\mathrm{non}}$ and the lower-left block *C* is numerically solved, $C=A\sqrt{I-u^{2}\Sigma^{2}}E^{†}$ with identity matrix *I*. The matrix elements *A*, *E*, and Σ are obtained from the singular value decomposition (SVD) of the operator $U_{\mathrm{non}}$: $U_{\mathrm{non}}=A\Sigma E^{†}$ [23,86], where Σ is a diagonal matrix containing the singular values, and *A*, *E* are unitary matrices.

To construct the full unitary operator *U*, we first perform QR decomposition on an associated ansatz matrix:

(A14) $$U^{\prime }=\left[\begin{array}{cc} uU_{\mathrm{non}} & I\\ C & I \end{array}\right],$$

where the right-hand blocks are simply taken to be identity matrices. To recover *U*, we can apply the QR decomposition to $U^{\prime }$, such as to obtain

(A15) $$U^{\prime }=UM,$$

where *U* ($UU^{†}=I$) is the desired unitary operator, and *M* is an upper triangular matrix.

Here, we present a concrete example to demonstrate how $U_{\mathrm{non}}$ can be executed from the *U* circuit. We can consider an initial composite state |ψ〉⊗|0〉, where |ψ〉 is the input state for physical problems, and |0〉 is for the ancilla qubit. The action of the unitary operator *U* on this state can be expressed as

(A16) $$U\left(|\psi \rangle ⊗|0\rangle \right)=\left(u\;U_{\mathrm{non}}|\psi \rangle \right)⊗|0\rangle +\left(C|\psi \rangle \right)⊗|1\rangle ,$$

where $U_{\mathrm{non}}$ denotes the desired non-unitary evolution. Then, we perform a projective measurement on the ancilla qubit. The post-selecting outcome with |0〉 is recorded, and the undesired branch associated with |1〉 is discarded. The resulting normalized state of the system qubits is

(A17) $$\frac{U_{\mathrm{non}}|\psi \rangle }{∥U_{\mathrm{non}}|\psi \rangle ∥}.$$

The implementation of this unitary matrix *U* can be achieved through variational circuits. Note that the Hamiltonian defined by Equation (A9) includes long-range couplings, so the optimization of variational circuits requires deep layers. As a result, the QITE method in this work is primarily applied to small-scale systems, where circuit depth and computational cost remain manageable. For example, the 9-qubit instance presented in the main text requires only two layers in the QAOA circuit, whereas the corresponding QITE circuit demands between 4 and 8 layers to achieve convergence. Despite the scaling challenges, larger instances, such as those with 20 and 30 qubits, can still be addressed using shallow QAOA circuits with just two layers.

For the optimization of QITE circuits, we set a convergence threshold of $C^{\mathrm{QITE}}<0.1$ (Equation (10)). This ensures that the evolved state closely approximates the ground state, and also keeps the circuit depth moderate and computationally manageable. As shown in Figure 7a, such imperfect convergence can give rise to a broad spread in the return error.

The classical compilation cost of QITE is illustrated in Figure A2. For a representative nine-qubit instance and a four-layer circuit, the compilation fidelity rises rapidly during the first approximately 600 objective evaluations and saturates near $F_{\mathrm{comp}}≃0.90$ after approximately 1200 evaluations. The optimization was extended to 4000 evaluations to confirm convergence.

## Appendix E. Quantum Annealing and Adiabatic Evolution

Quantum annealing is another approach designed to solve optimization and minimization problems [87,88,89]. This method is based on the adiabatic evolution of a quantum system governed by a time-dependent Hamiltonian that interpolates between an initial Hamiltonian $H_{i}$ and the target Hamiltonian $H_{p}$. This evolution is described by: $H\left(\lambda \left(t\right)\right)=\left(1-\lambda \left(t\right)\right)H_{i}+\lambda \left(t\right)H_{p},$ where λ(t) is a time-dependent annealing parameter satisfying the boundary conditions λ(0)=0,λ(T)=1. The system is initially prepared in the ground state of $H_{i}$ and evolves under $H_{ad}\left(\lambda \left(t\right)\right)$, with the target Hamiltonian gradually turned on. According to the quantum adiabatic theorem, if the evolution proceeds slowly enough, the system remains in its instantaneous ground state and eventually transitions to the ground state of $H_{p}$.

In general, quantum annealing can be naturally implemented on analog quantum simulators, where the evolution on such quantum platforms closely follows a continuous-time annealing schedule. However, the simulator employed in this work is digital, and emulating quantum annealing dynamics on such a platform requires extremely deep quantum circuits to Trotterize the continuous evolution. These deep circuits are highly susceptible to noise and decoherence. Given this, we do not include quantum annealing in our performance benchmarks.

## Appendix F. Additional Results

Here, we present additional results to demonstrate that the noiseless optimization of QAOA can reach reasonable convergence. These results are presented in Figure A3, for the four instances shown in Figure 5 (main text). These results clearly demonstrate that, in the absence of noise, QAOA can converge toward the ground state, successfully identifying the optimal bitstring for each instance. Note that for different instances, the corresponding Hamiltonian might also contain different parameters corresponding to different instances of generated asset data, leading to the convergence to distinctive bitstrings.

## Appendix G. Circuit-Depth and Two-Qubit-Error Estimate

Because QAOA and QITE were executed using different classical and quantum environments, their classical runtime and total wall-clock time cannot be compared directly in a hardware-independent manner. We, therefore, restrict the resource estimate to the circuit depth and accumulated two-qubit-gate error, which can be evaluated consistently from the circuit architecture used in this work.

For a variational circuit acting on *q* qubits with *d* layers, each nearest-neighbor entangling sublayer contains approximately ⌊q/2⌋ parallel ECR gates. Before hardware-dependent transpilation, the two-qubit-gate count is, therefore,

(A18) $$N_{\mathrm{ECR}}=d\left\lfloor \frac{q}{2}\right\rfloor ,$$

while the logical two-qubit-gate depth is *d* because the non-overlapping ECR gates within each layer can be executed in parallel. For the nine-qubit QAOA calculations, q=9 and d=2, giving

(A19) $$N_{\mathrm{ECR}}^{\mathrm{QAOA}}=8,\;D_{2q}^{\mathrm{QAOA}}=2.$$

For QITE, the circuit acts on nine system qubits and one ancilla, giving q=10. The four- to eight-layer circuits used in this work, therefore, have

(A20) $$N_{\mathrm{ECR}}^{\mathrm{QITE}}=20–40,\;D_{2q}^{\mathrm{QITE}}=4–8.$$

If each ECR gate has an average error probability $\epsilon_{2q}$, the probability of encountering at least one two-qubit error can be estimated, under an independent-error approximation, as

(A21) $$P_{2q}≃1-{\left(1-\epsilon_{2q}\right)}^{N_{\mathrm{ECR}}}.$$

Using the mean value $\epsilon_{2q}≃0.007$ measured for the selected IBM qubit chain gives

(A22) $$P_{2q}^{\mathrm{QAOA}}≃1-{\left(1-0.007\right)}^{8}≃5.5\%,$$

and

(A23) $$P_{2q}^{\mathrm{QITE}}≃1-{\left(1-0.007\right)}^{20–40}≃13\%–25\%.$$

These estimates show that an individual QAOA circuit is shallower and has lower accumulated two-qubit-gate exposure than the QITE circuits used here. Nevertheless, QAOA repeatedly evaluates noisy circuits during variational optimization, so even relatively small circuit-level errors can perturb the cost function and alter the optimization trajectory. By contrast, QITE requires a deeper final circuit but avoids performing the complete variational optimization loop on quantum hardware.

## Appendix H. Estimated QAOA Workload on Quantum Hardware

A complete QAOA implementation requires a sequential quantum–classical optimization loop. For each cost-function evaluation, the parameterized circuit is executed, the computational-basis outcomes are collected, the energy is reconstructed, and the classical optimizer generates the parameters for the next circuit. Since each update depends on the preceding measurement result, the full set of circuits cannot be submitted in advance. For the nine-qubit instances considered in this work, the optimization typically requires approximately $N_{\mathrm{eval}}=600$–1000 cost-function evaluations. If $N_{s}$ shots are used for each evaluation, the total number of hardware shots is

(A24) $$N_{\mathrm{shot}}^{\mathrm{QAOA}}=N_{\mathrm{eval}}N_{s}.$$

For example, using $N_{s}=10^{4}$ shots per evaluation gives a total workload of approximately

(A25) $$N_{\mathrm{shot}}^{\mathrm{QAOA}}≃6\times 10^{6}–10^{7}$$

shots for a single portfolio instance. Repeating the calculation for four instances would increase this requirement to approximately $2.4\times 10^{7}$–$4.0\times 10^{7}$ shots, before including independent optimization restarts, calibration circuits, or error-mitigation overhead.

The wall-clock time of a hardware QAOA optimization can be decomposed as

(A26) $$T_{\mathrm{QAOA}}=\sum_{k=1}^{N_{\mathrm{eval}}}\left(T_{\mathrm{queue}}^{\left(k\right)}+T_{\mathrm{QPU}}^{\left(k\right)}+T_{\mathrm{classical}}^{\left(k\right)}+T_{\mathrm{communication}}^{\left(k\right)}\right),$$

where $N_{\mathrm{eval}}$ is the number of sequential cost-function evaluations. The QPU usage for one evaluation can be approximately written as

(A27) $$T_{\mathrm{QPU}}^{\left(k\right)}≃T_{\mathrm{overhead}}+N_{s}\left(T_{\mathrm{rep}}+T_{\mathrm{circ}}\right),$$

where $N_{s}$ is the number of shots, $T_{\mathrm{rep}}$ is the repetition delay between circuit executions, and $T_{\mathrm{circ}}$ is the duration of one transpiled circuit. For current IBM Quantum workloads, a baseline estimate uses an overhead of approximately 2s per submitted sub-job and approximately 0.35ms per circuit execution, including a typical repetition delay and circuit duration. For the nine-qubit QAOA calculations considered here, $N_{\mathrm{eval}}≃600$–1000. Taking $N_{s}=10^{4}$ shots per cost-function evaluation gives $N_{\mathrm{exec}}=N_{\mathrm{eval}}N_{s}≃6\times 10^{6}–10^{7}$ individual circuit executions. Because the optimization is sequential, each cost-function evaluation must be followed by a classical parameter update before the next circuit can be submitted. Treating each evaluation as one sub-job gives the baseline QPU-usage estimate

(A28) $$\begin{array}{cc} T_{\mathrm{QPU}}^{\mathrm{QAOA}} & ≃N_{\mathrm{eval}}\left[2\;s+0.00035\;s\times 10^{4}\right]\\ \; & ≃3.3\times 10^{3}–5.5\times 10^{3}\;s, \end{array}$$

corresponding to approximately 55–92 min of QPU usage for a single portfolio instance. Repeating the calculation for the four representative instances in Figure 3 would require approximately 3.7–6.1 h of QPU usage, before including independent optimizer restarts, calibration circuits, or error-mitigation overhead.

The actual wall-clock time can be considerably longer. If the classical optimization and communication between two successive evaluations require an average time $T_{\mathrm{gap}}$, the additional time is

(A29) $$T_{\mathrm{feedback}}=N_{\mathrm{eval}}T_{\mathrm{gap}}.$$

For example, even an average feedback delay of 1s adds approximately 10–17 min per instance, while a delay of 5s adds approximately 50–83 min. In ordinary job mode, repeated queue delays may increase the total elapsed time much further and are not reliably predictable. Thus, even for the shallow nine-qubit circuits used here, the repeated hardware-in-the-loop optimization makes a comprehensive QAOA benchmark substantially more expensive than executing a single offline-compiled QITE circuit.

## Appendix I. Energy-Gap Analysis and Choice of QAOA Shot Number

To estimate the sampling resolution required for the QAOA calculations, we analyze the exact spectrum of the diagonal portfolio Ising Hamiltonian. Because all terms are diagonal in the computational basis, the energy of each bitstring can be evaluated directly. After sorting the distinct energies as $E_{0}<E_{1}<E_{2}<\cdots ,$ we define the adjacent level gaps

(A30) $$\Delta_{k}=E_{k+1}-E_{k}.$$

Since the absolute energy scale varies between portfolio instances, the gaps are normalized by the full spectral width,

(A31) $${\tilde{\Delta }}_{k}=\frac{E_{k+1}-E_{k}}{E_{\max}-E_{\min}}.$$

The finite sampling resolution associated with $N_{\mathrm{shot}}$ measurements is taken to scale as $1/N_{\mathrm{shot}}$. We, therefore, require this resolution to be smaller than the normalized energy gap by a safety factor *s*,

(A32) $$\frac{1}{N_{\mathrm{shot}}}\leq \frac{{\tilde{\Delta }}_{k}}{s}.$$

The corresponding minimum shot number is

(A33) $$N_{\mathrm{shot}}^{\left(k\right)}=\left\lceil \frac{s}{{\tilde{\Delta }}_{k}}\right\rceil .$$

In the calculations shown here, we use s=10.

Figure A4 presents this analysis for representative portfolio instance 52. The black circles show the normalized adjacent gaps among the first ten distinct energy levels, while the dashed lines show $s/N_{\mathrm{shot}}$ for several candidate shot numbers. All displayed gaps lie below the threshold corresponding to $N_{\mathrm{shot}}$ = 40,000. Therefore, 40,000 shots are insufficient to resolve every neighboring low-energy level of this instance under the criterion in Equation (A32).
